# Free Convection Nanofluid Flow in the Stagnation-Point Region of a Three-Dimensional Body

**DOI:** 10.1155/2014/158269

**Published:** 2014-07-08

**Authors:** Umer Farooq, Hang Xu

**Affiliations:** State Key Laboratory of Ocean Engineering, School of Naval Architecture, Ocean and Civil Engineering, Shanghai Jiao Tong University, Shanghai 200240, China

## Abstract

Analytical results are presented for a steady three-dimensional free convection flow in the stagnation point region over a general curved isothermal surface placed in a nanofluid. The momentum equations in *x*- and *y*-directions, energy balance equation, and nanoparticle concentration equation are reduced to a set of four fully coupled nonlinear differential equations under appropriate similarity transformations. The well known technique optimal homotopy analysis method (OHAM) is used to obtain the exact solution explicitly, whose convergence is then checked in detail. Besides, the effects of the physical parameters, such as the Lewis number, the Brownian motion parameter, the thermophoresis parameter, and the buoyancy ratio on the profiles of velocities, temperature, and concentration, are studied and discussed. Furthermore the local skin friction coefficients in *x*- and *y*-directions, the local Nusselt number, and the local Sherwood number are examined for various values of the physical parameters.

## 1. Introduction

The stagnation point is defined as the point on the surface of objects in the flow field where the fluid is brought at rest by the object. The problem of flow and heat transfer at a general three-dimensional stagnation-point region has important applications in many manufacturing processes in petrochemical industries, the aerodynamic of plastic sheet, solar central receivers exposed to wind currents, and so forth. The boundary layer flows near a three-dimensional stagnation point of attachment on an isothermal surface have been examined several times in the past. Howarth [1], Davey and Schofield [2] discussed the solutions of boundary layer flow near the stagnation point on a general three-dimensional surface. Poots [3] formulated the boundary-layer equations for the free convection flow at three-dimensional lower stagnation point on a general curved isothermal surface. Banks [4] concluded that the three-dimensional solution can be exhibited at a two-dimensional stagnation point for a Prandtl number Pr = 0.72. Further he investigated at infinitely large Prandtl number Pr = *∞* the three-dimensional problem which can be reduced to the two-dimensional case. Banks [5] then extended the results of Poots [3] for negative values of *c* (referred to as dual solution) corresponding to saddle points of attachment. Gorla et al. [6] explored dual solutions by a numerical procedure in the investigation of nonnewtonian power-law type fluids near a three-dimensional stagnation point. Ingham et al. [7] considered unsteady free convection flow near a three-dimensional stagnation point of attachment on an isothermal surface. Pop and Merkin [8] validated that the form of the three-dimensional free convection near a stagnation point on an isothermal surface depends on *c* = *b*/*a*, the ratio of the principle curvature of the body at a stagnation point. Merkin and Mahmood [9] investigated free convection boundary layer on a vertical plate with prescribed surface heat flux. Takhar et al. [10] studied unsteady free convection boundary layer flow in the forward stagnation-point region of a sphere, which is rotating with time dependent angular velocity in an ambient fluid. Slaouti et al. [11] described the momentum and the thermal boundary layer characteristics for unsteady free convection flow in the stagnation-point region of a heated three-dimensional body. Harris et al. [12] examined the free convection boundary layer flow near the lower stagnation point of a cylindrical body. Xu et al. [13] presented series solutions of unsteady free convection flow in the stagnation-point region of a three-dimensional body. Singh et al. [14] extended the work of Xu et al. [13] to the mass transfer case. Admon et al. [15] presented flow and heat transfer analysis for the unsteady free convection flow near the stagnation point of a three-dimensional body.

Nanoparticles are defined to be an object with at least one dimension smaller than 100 nanometer. When particle is such small and the area of the particle which got the surface is much higher than its weight, that makes particle having new and exciting properties. The addition of nanoparticles in a clear fluid like water and a solution where the suspension of nanoparticles can be stabilized; then, this setup is called nanofluids. Choi [16] is the first among the others to use the term nanofluids. The purpose of the nanofluids is to intensify the heat transfer properties and the development of the nanofluid is potentially for several applications such as cooling of electronics and cooling of data center where a computer eject a lot of heat, coolants, and so forth. The in-depth study on nanofluids can be found in the book by Das et al. [17], in the review paper by Wang and Mujumdar [18], Yang et al. [19], and Jahani et al. [20]. Kuzetsov and Nield [21] used Buogiorno [22] model to investigate a natural convection flow of a nanofluid over a vertical plate. They identified governing parameters for the transport process, namely, a buoyancy-ratio number *N*_*r*_, a thermophoresis parameter *N*_*t*_, a Brownian number *N*_*b*_, and a Lewis number Le. Recently the stagnation-point flow of a nanofluid has drawn considerable attention and a significant amount of research is in progress. Bachok et al. [23] studied the effects of nanoparticle volume fraction parameter and the ratio of gradients of velocities on the flow and heat transfer for the steady three-dimensional stagnation-point flow in a nanofluid. Bachok et al. [24] investigated two-dimensional stagnation-point flow of a nanofluid over a stretching/shrinking sheet. Alsaedi et al. [25] examined the influence of heat generation/absorption on the stagnation-point flow of nanofluids towards a linear stretching surface. Mustafa et al. [26] extended the work of Ibrahim et al. [27] with the inclusion of the combined effects of buoyancy force and convective heating.

In this paper the three-dimensional laminar free convection stagnation-point flow of a nanofluid on a general curved isothermal surface is considered. The conservation equations embodying the mass, the momentum, and the energy as well as the nanoparticle volume fraction are reduced to a set of four fully coupled nonlinear equations. Exact analytical approximations are obtained for the velocity, temperature, and concentration profile as well as for the local skin friction coefficients, the local Nusselt number, and the local Sherwood number by means of the OHAM technique [28]. A new kind of error estimation technique is defined and used to evaluate the convergence of the solution series. It should be noted that two different approaches can be used to choose the optimal values of the convergence-control parameters. One approach is the *ℏ*-curve method which reflects the variation of *ℏ* with a fixed unknown functional value (for an example, *f*′′(0)) given by a certain order of HAM approximation. Other technique is the minimum error method [29], in which we can select the “best” value of the convergence-control parameters by the minimum errors of the whole equations. On the other hand, the explicit solutions are given based on the HAM technique (for details see Appendix B). Besides The effects of the physical parameters such as Le, *N*_*b*_, *N*_*t*_, and *N*_*r*_ on profiles of velocities, temperature, and concentration as well as the important quantities such as the local skin friction coefficients, the local Nusselt number, and the local Sherwood number are explored in detail.

## 2. Problem Formulation

**Figure 1:**
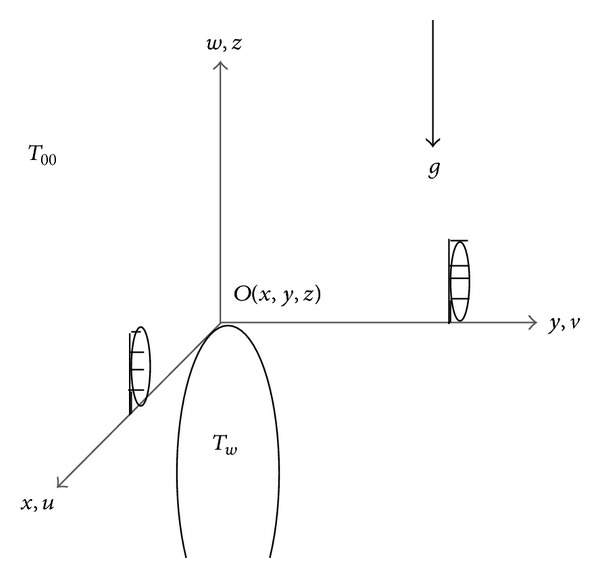
Physical configuration.

Consider steady, viscous, laminar, incompressible, and free convection nanofluid flow in the stagnation-point region of a three-dimensional body. A Cartesian coordinate system (*x*, *y*, *z*) is chosen with the origin *O* at the stagnation point as shown in the Figure 1, where *x*- and *y*-coordinates are measured along the body surface while the *z*-coordinate is measured normal to the body surface. We assume that the flow is steady, the temperature and concentration are *T*_*w*_ and *C*_*w*_ at the surface, and the temperature and concentration for the ambient fluid is *T*_*∞*_ and *C*_*∞*_ and the gravitational acceleration is *g*, respectively. Under these assumptions the governing equations of continuity, momentum, energy, and nanoparticle volume fraction are as follows.

Continuity Equation

(1) $$\begin{array}{c} \frac{\partial u}{\partial x}+\frac{\partial v}{\partial y}+\frac{\partial w}{\partial z}=0. \end{array}$$

Momentum equations for *x*- and *y*-components

(2) u∂u∂x+v∂u∂y+w∂u∂z =−1ρf∞∂p∂x+ν(∂2u∂x2+∂2u∂y2+∂2u∂z2)  +[(1−C∞)β(T−T∞)−ρp−ρf∞ρf∞(C−C∞)]gax,u∂v∂x+v∂v∂y+w∂v∂z =−1ρf∞∂p∂y+ν(∂2v∂x2+∂2v∂y2+∂2v∂z2)  +[(1−C∞)β(T−T∞)−ρp−ρf∞ρf∞(C−C∞)]gby.

Thermal energy equation

(3) u∂T∂x+v∂T∂y+w∂T∂z =α(∂2T∂x2+∂2T∂y2+∂2T∂z2)  +τ{DB(∂C∂x∂T∂x+∂C∂y∂T∂y+∂C∂z∂T∂z)    +(DTT∞)[(∂T∂x)2+(∂T∂y)2+(∂T∂z)2]}.

Nanoparticle volume fraction equation

(4) u∂C∂x+v∂C∂y+w∂C∂z=DB(∂2C∂x2+∂2C∂y2+∂2C∂z2)+(DTT∞)(∂2T∂x2+∂2T∂y2+∂2T∂z2).

In the above equations, *u*,  *v*, and  *w* are the *x*-component, *y*-component, and *z*-componen, respectively, of the fluid velocity with origin *O*; *ν* is the kinematic viscosity; *β* is the coefficient of thermal expansion; *C* is the nanoparticle volume fraction; *D*_*B*_ is the Brownian diffusion coefficient; *D*_*T*_ is the thermophoretic diffusion coefficient; *τ* is the heat capacity ratio. Subscripts *p* and *f*, respectively, denote the nanoparticles and the base fluid. The two functions *a* and *b* are the principal curvatures of the body at the stagnation point. The corresponding boundary conditions are

(5) $$\begin{array}{c} u=v=w=0,\;T=T_{w},\;C=C_{w}\;\text{at}\;\;z=0,\\ u⟶0,\;v⟶0,\;T⟶T_{\infty },\;C⟶C_{\infty }\;\text{as}\;\;\eta ⟶\infty . \end{array}$$

We now introduce the similarity transformations as follows:

(6) $$\begin{array}{c} \eta =\text{G}{\text{r}}^{1/4}az,\;\;u=\nu a^{2}\text{G}{\text{r}}^{1/2}xf^{\prime },\;\;v=\nu a^{2}\text{G}{\text{r}}^{1/2}cys^{\prime },\\ w=-\nu a\text{G}{\text{r}}^{1/4}\left(f+s\right),\;\;\theta \left(\eta \right)=\frac{T-T_{\infty }}{T_{w}-T_{\infty }},\;\\ \phi \left(\eta \right)=\frac{C-C_{\infty }}{C_{w}-C_{\infty }}. \end{array}$$

Substituting (6) into (1)–(5), the nondimensionalized governing equations take the forms

(7) $$\begin{array}{c} f^{\prime \prime \prime }+\left(f+cs\right)f^{\prime \prime }-{\left(f^{\prime }\right)}^{2}+\theta -\frac{N_{r}}{\text{Pr}\;\text{Gr}}\phi =0, \end{array}$$

(8) $$\begin{array}{c} s^{\prime \prime \prime }+\left(f+cs\right)s^{\prime \prime }-c{\left(s^{\prime }\right)}^{2}+\theta -\frac{N_{r}}{\text{Pr}\;\text{Gr}}\phi =0, \end{array}$$

(9) $$\begin{array}{c} \frac{1}{\text{Pr}}\theta^{\prime \prime }+\left(f+cs\right)\theta^{\prime }+\frac{N_{b}}{\text{Pr}}\theta^{\prime }\phi^{\prime }+\frac{N_{t}}{\text{Pr}}{\left(\theta^{\prime }\right)}^{2}=0, \end{array}$$

(10) $$\begin{array}{c} \phi^{\prime \prime }+\frac{N_{t}}{N_{b}}\theta^{\prime \prime }+\text{Le}\;\text{Pr}\left(f+cs\right)\phi^{\prime }=0. \end{array}$$

In the above equations, the parameters Pr, *N*_*b*_, *N*_*r*_, and *N*_*t*_ are the Prandtl number, the Brownian motion parameter, the buoyancy parameter, and the thermophoresis parameter, respectively, which are defined by

(11) $$\begin{array}{c} \text{Pr}=\frac{\nu }{\alpha },\;\;N_{b}=\frac{\tau D_{B}\left(C_{w}-C_{\infty }\right)}{\alpha },\\ N_{r}=\frac{\left(\rho_{p}-\rho_{f_{\infty }}\right)\left(C_{w}-C_{\infty }\right)g}{\mu \alpha a^{3}},\;\;N_{t}=\tau \frac{D_{T}}{T_{\infty }}\frac{T_{w}-T_{\infty }}{\alpha }, \end{array}$$

where *α* is the thermal conductivity, Gr and Le are the Grashof number and Lewis number, respectively. They are defined as

(12) $$\begin{array}{c} \text{Gr}=\frac{\left(1-C_{\infty }\right)\beta g\left(T_{w}-T_{\infty }\right)}{\nu^{2}a^{3}},\;\;\text{Le}=\frac{\alpha }{D_{B}},\;\;c=\frac{b}{a}. \end{array}$$

The boundary conditions in nondimensional form are

(13) $$\begin{array}{c} f\left(0\right)=0,\;\;f^{\prime }\left(0\right)=0,\;\;f^{\prime }\left(\infty \right)=0,\\ s\left(0\right)=0,\;\;s^{\prime }\left(0\right)=0,\;\;s^{\prime }\left(\infty \right)=0,\\ \theta \left(0\right)=1,\;\;\theta \left(\infty \right)=0,\\ \phi \left(0\right)=1,\;\;\phi \left(\infty \right)=0. \end{array}$$

The quantities of physical interest such as the local skin friction coefficients *C*_*fx*_ and *C*_*fy*_, along the *x*- and *y*-directions respectively and the local Nusselt number and the local Sherwood number, which are defined as

(14) $$\begin{array}{c} C_{fx}=\frac{\tau_{wx}}{\rho u_{x}^{2}},\;\;C_{fy}=\frac{\tau_{wy}}{\rho u_{y}^{2}},\\ {\text{Nu}}_{x}=\frac{xq}{K\left(T_{w}-T_{\infty }\right)},\;\;{\text{Sh}}_{x}=\frac{xj}{D_{B}\left(C_{w}-C_{\infty }\right)}, \end{array}$$

where *u*_*x*_ = *νa* ^2^Gr^1/2^ *x* and *u*_*y*_ = *νa* ^2^Gr^1/2^ *cy* are the local reference velocities, and *τ*_*wx*_, *τ*_*wy*_, *q*_*w*_, and *j*_*w*_ are the local wall skin friction, the local heat flux, and the mass flux from the surface defined by

(15) $$\begin{array}{c} \tau_{wx}=\mu {\left(\frac{\partial u}{\partial z}\right)}_{z=0},\;\;\tau_{wy}=\mu {\left(\frac{\partial v}{\partial z}\right)}_{z=0},\\ q_{w}=-K{\left(\frac{\partial T}{\partial z}\right)}_{z=0},\;\;j_{w}=-D_{B}{\left(\frac{\partial C}{\partial Z}\right)}_{z=0}. \end{array}$$

Substituting (6) in (15) the nondimensional skin friction coefficients, the Nusselt number, and the Sherwood number are as follows:

(16) $$\begin{array}{c} \text{R}{\text{e}}_{x}^{1/2}C_{fx}=f^{\prime \prime }\left(0\right),\;\;\text{R}{\text{e}}_{y}^{1/2}C_{fy}=s^{\prime \prime }\left(0\right),\\ \text{R}{\text{e}}_{x}^{-1/2}{\text{Nu}}_{x}=-\theta^{\prime }\left(0\right),\;\;\text{R}{\text{e}}_{x}^{-1/2}\text{S}{\text{h}}_{x}=-\phi^{\prime }\left(0\right), \end{array}$$

where Re_*x*_ = *u*_*x*_ *x*/*ν* and Re_*y*_ = *u*_*y*_ *x*/*ν* are the local Reynolds number in *x*- and *y*-directions, respectively. Note that the local Nusselt number and the local Sherwood number have the same values in both *x*- and *y*-directions, so we only list them in *x*-direction.

## 3. Convergence Criterion

### 3.1. Exact Residual Error

It should be emphasized that *f*_*k*_(*η*), *s*_*k*_(*η*), *θ*_*k*_(*η*), and *ϕ*_*k*_(*η*) as defined in Appendix A contain unknown convergence-control parameters *c*_0_^*f*^, *c*_0_^*s*^, *c*_0_^*θ*^, and *c*_0_^*ϕ*^, which determine the convergence region and rate of the homotopy-series solutions. To find out the optimal values of *c*_0_^*f*^, *c*_0_^*s*^, *c*_0_^*θ*^, and *c*_0_^*ϕ*^, first we define the exact squared residual errors at the *k*th-order of approximations as follows:

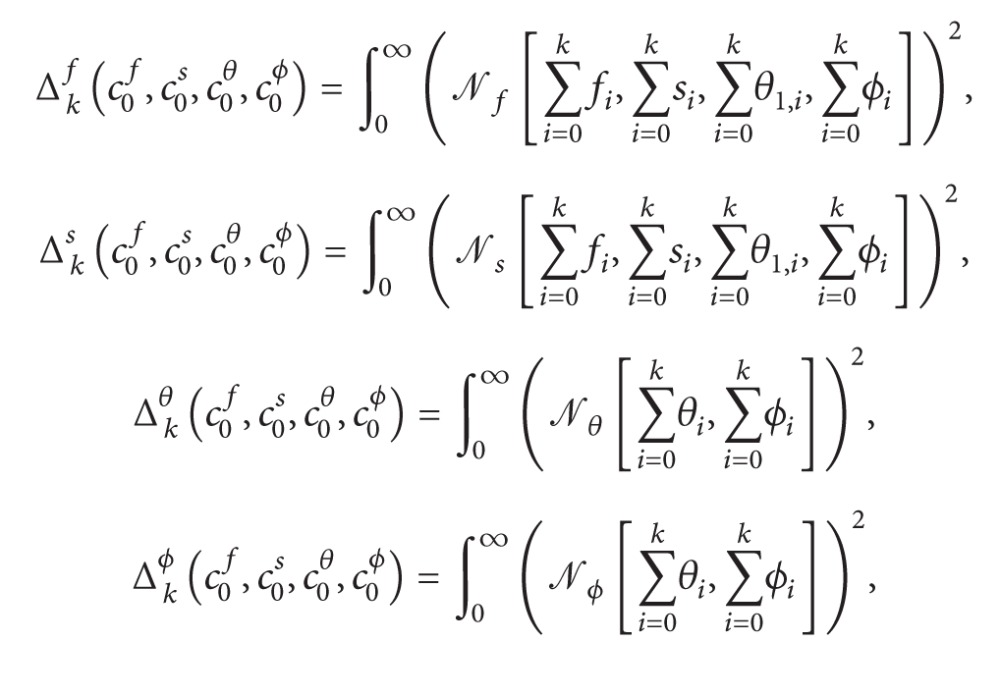
(17)

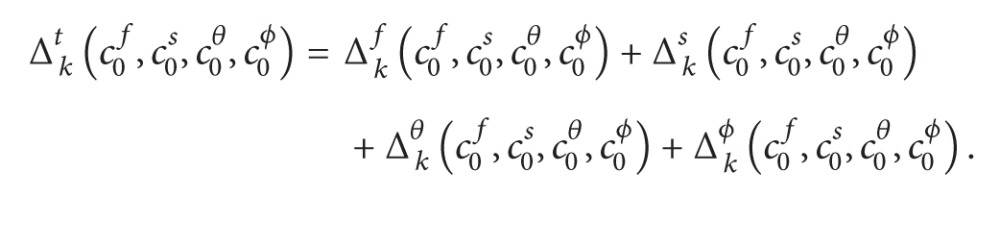
(18)
Note that Δ_*k*_^*t*^ contains all the unknown convergence-control parameters *c*_0_^*f*^, *c*_0_^*s*^, *c*_0_^*θ*^, and *c*_0_^*ϕ*^. Obviously, the more quickly Δ_*k*_^*t*^ decreases to zero, the faster the corresponding homotopy-series solutions converge. Thus, at the given order of approximation *k*, the corresponding optimal values of the convergence-control parameters are given by the minimum of Δ_*k*_^*t*^, corresponding to a set of four nonlinear algebraic equations

(19) $$\begin{array}{c} \frac{\partial \Delta_{k}^{t}}{\partial c_{0}^{f}}=0,\;\;\frac{\partial \Delta_{k}^{t}}{\partial c_{0}^{s}}=0,\;\;\frac{\partial \Delta_{k}^{t}}{\partial c_{0}^{\theta }}=0,\;\;\frac{\partial \Delta_{k}^{t}}{\partial c_{0}^{\phi }}=0. \end{array}$$

However, Δ_*k*_^*t*^ defined by (18) takes too much CPU time to calculate even if the order of approximation is not very high.

### 3.2. Average Squared Residual Error

Thus, to greatly decrease the CPU time, it is used here the so-called average squared residual error defined by

(20) Ekf(c0f,c0s,c0θ,c0ϕ) =1N+1∑j=0N[Nf(∑i=0kfi,∑i=0ksi,∑i=0kθi,∑i=0kϕi)|η=jδη]2,Eks(c0f,c0s,c0θ,c0ϕ) =1N+1∑j=0N[Ns(∑i=0kfi,∑i=0ksi,∑i=0kθi,∑i=0kϕi)|η=jδη]2,Ekθ(c0f,c0s,c0θ,c0ϕ)=1N+1∑j=0N[Nθ(∑i=0kθi,∑i=0kϕi)|η=jδη]2,Ekϕ(c0f,c0s,c0θ,c0ϕ)=1N+1∑j=0N[Nϕ(∑i=0kθi,∑i=0kϕi)|η=jδη]2,

where *N* is an integer. It is reasonable to set *N* = 80 and *δη* = 0.1. we define

(21) Ekt(c0f,c0s,c0θ,c0ϕ) =Ekf(c0f,c0s,c0θ,c0ϕ)+Eks(c0f,c0s,c0θ,c0ϕ)  +Ekθ(c0f,c0s,c0θ,c0ϕ)+Ekϕ(c0f,c0s,c0θ,c0ϕ),

where *E*_*k*_^*t*^ is the total average squared residual error. Obviously, the smaller the value of *E*_*k*_^*t*^(*c*_0_^*f*^, *c*_0_^*s*^, *c*_0_^*θ*^, *c*_0_^*ϕ*^) for given iteration *k*, the better the approximation. The OHAM series solutions which greatly depend on optimal values of convergence-control parameters can be obtained accurately by the proper choice of convergence-control parameters.

### 3.3. Choice of Convergence-Control Parameters

In this subsection we will explain the procedure to obtain the optimal convergence-control parameters. The optimal values for the convergence-control parameters can be obtained by the minimum of total average squared residual error. The total average squared residual error *E*_*k*_^*t*^(*c*_0_^*f*^, *c*_0_^*s*^, *c*_0_^*θ*^, *c*_0_^*ϕ*^) is minimized by using symbolic computation software *Mathematica*. It is clear the smaller the value of total averaged squared residual error for a given *k* the better the approximation. We directly employ the command* Minimize* at any order of iteration, say “*k*,” to obtain the corresponding global optimal convergence-control parameters. Let us assume Pr = Le = Gr = 1.0, *c* = 0.5, *N*_*b*_ = *N*_*t*_ = *N*_*r*_ = 0.1, and the convergence-control parameters are unknown. The corresponding squared residual errors at different orders of iterations are shown in Table 1. It can be seen as we increase the order that the total average squared residual error is decreasing. Physically the convergence-control parameters are just the artificial parameters introduced in the high order deformation equations to obtain the convergent results. Since the convergent-control parameters are unknown therefore the CPU takes too much computation time. Therefore it is reasonable to choose optimal values of the convergence-control parameters corresponding to 5-order OHAM iteration. Using fifth order OHAM iteration the OHAM approximations at different orders of iterations are shown in Table 2. The results in Table 2 demonstrate the reliability of OHAM series solutions.

## 4. Results and Discussion

Analytical approximations with the high precision for velocity profile along *x*- and *y*-directions, temperature distribution, and concentration profile, the local skin friction coefficients along *x*- and *y*-directions, the local Nusselt number, and the local sherwood number are plotted in Figures 2–13 for various values of the physical parameters Le, *N*_*r*_, *N*_*t*_, *N*_*b*_, and *c*.

**Figure 2:**
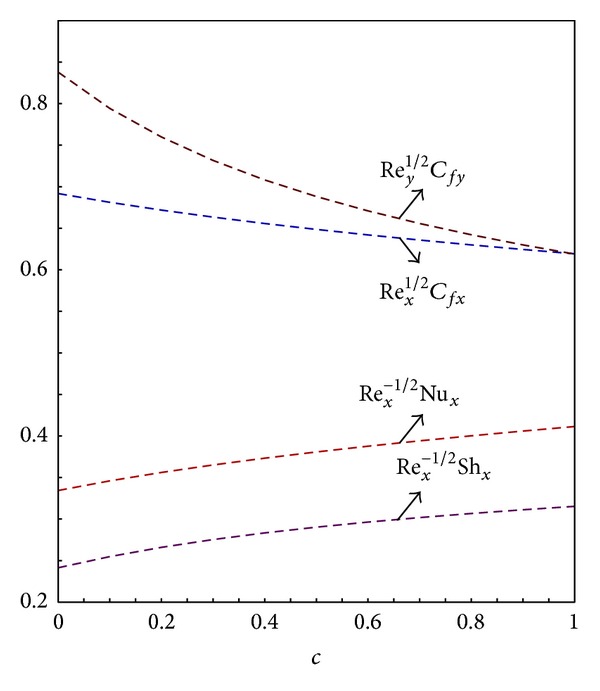
Graphs of skin friction coefficients, Nusselt and Sherwood numbers. *N*_*b*_ = *N*_*t*_ = *N*_*r*_ = 0.1 and Le = 1.0.

Figure 2 depicts the graphs of the local skin friction coefficients, the local Nusselt number, and the local Sherwood number for various values of *c* keeping the other parameters such as Pr = Gr = Le = 1.0, *N*_*b*_ = *N*_*t*_ = *N*_*r*_ = 0.1. Since we have assumed that *a* and *b* are positive and most shapes of practical interest lies between cylinder (*c* = 0) and sphere (*c* = 1) therefore it is reasonable to consider the values of *c* between 0 and 1 however the current method can also be applied to generate the solutions for (1 ≤ *c* ≤ *∞*). The increase in the values of *c* increases parameter *b* (the curvature of the body in the *x* = 0 plane) and decreases the parameter *a* (the curvature of the body in the *y* = 0 plane) and also the impact of the increase in the values of *c* is to decrease the shear stress because of more streamlined flow. It can be seen in Figure 2 that the skin friction coefficients attains their maximum values in the stagnation point under the boundary layer effects and the increase in the value of *c* reduces the skin friction. Since the increase in the values *c* is due to the increase in the parameter *b* hence the decrease in the skin friction is more pronounced in *y*-direction. The analysis of the parameter *c* on the heat and mass transfer shows the increase in the values of *c* will increase the local Nusselt number and the local Sherwood number.

**Figure 3:**
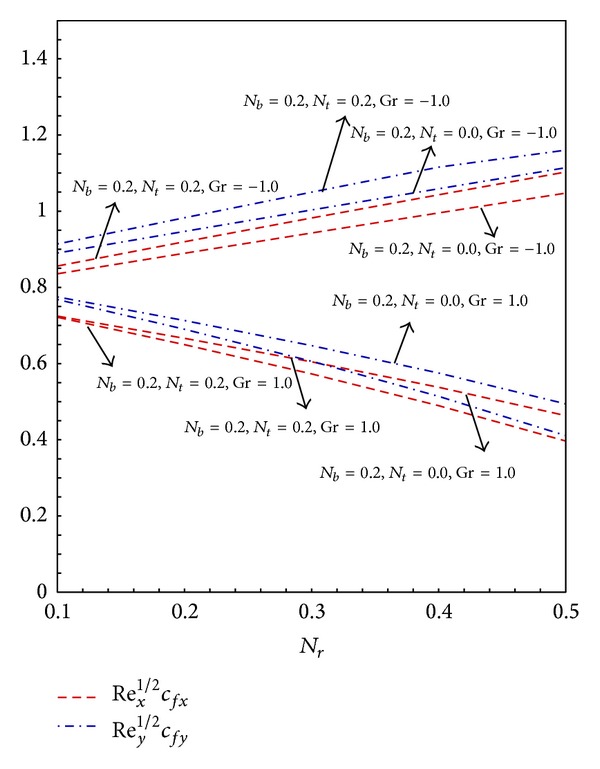
Graphs of skin friction coefficient along *x*- and *y*-direction for varying *N*_*r*_.

**Figure 4:**
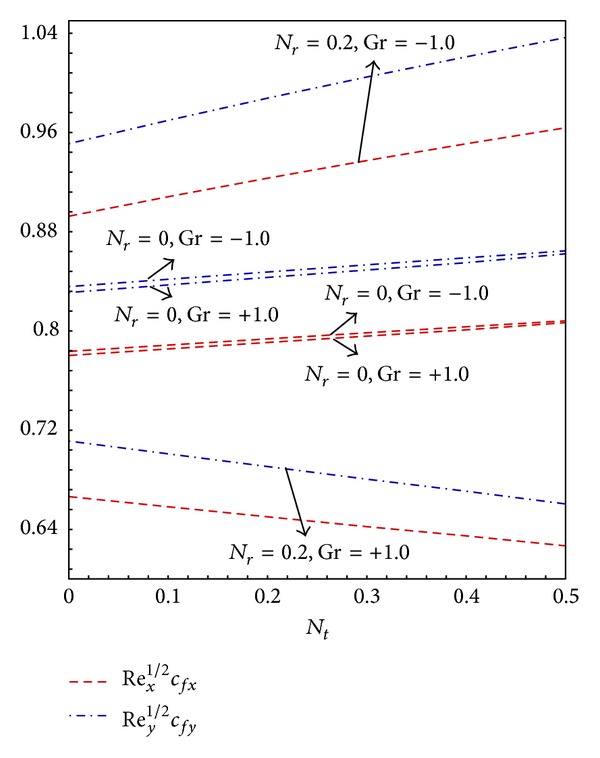
Graphs of skin friction coefficient along *x*- and *y*-direction for *N*_*b*_ = 0.2 and varying *N*_*t*_.

**Figure 5:**
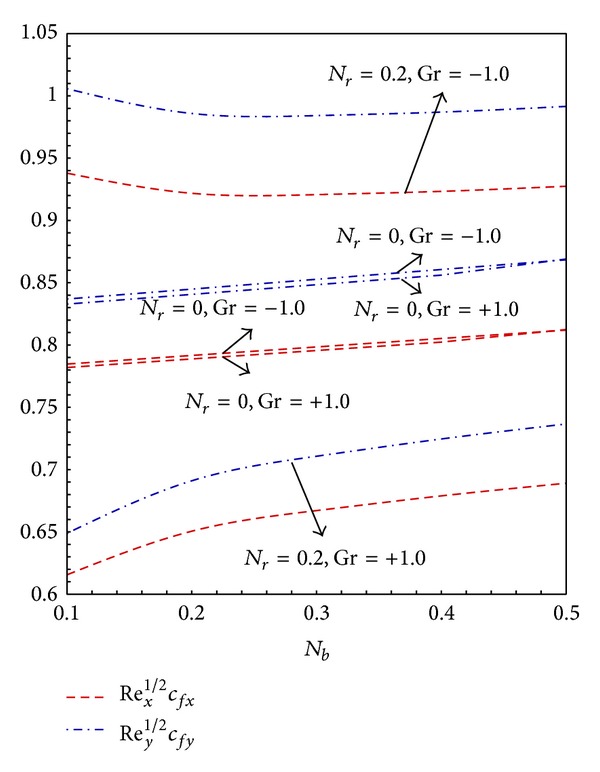
Graphs of skin friction coefficient along *x*- and *y*-direction for *N*_*t*_ = 0.2 and varying *N*_*b*_.

Figure 3 reveals the effects of *N*_*r*_ and *N*_*t*_ on the local skin friction for both buoyancy assisted Gr = 1.0 and buoyancy opposing flow Gr = −1.0. For buoyancy assisted flow the temperature at the stagnation point *T*_*w*_ is greater than the ambient fluid temperature *T*_*∞*_, that is, *T*_*w*_ > *T*_*∞*_. The increase in the values of *N*_*r*_ will increase the density and nanoparticles mass differences; thus, the fluid becomes more dense which opposes the transport phenomenon. It can be seen in Figure 3 the skin friction coefficient decreases with the increase in the values of *N*_*r*_. For buoyancy opposing case the ambient fluid temperature is higher, that is, *T*_*∞*_ > *T*_*w*_ the increase in the values of *N*_*r*_ will result in the decrease in the density and mass fraction nanoparticles differences hence the fluid is less dense. For buoyancy opposing case this can be seen in Figure 3 the increase in the values of *N*_*r*_ increases the skin friction coefficients. Further it can be seen for buoyancy assisted flow the increase in the values of *N*_*t*_ increases the temperature difference which increases the density difference, the ambient fluid is more dense hence the skin friction along *x*- and *y*-directions decreases while quite the opposite is true for buoyancy opposing flow.  Figure 4 describes the effects of *N*_*t*_ on the local skin friction coefficients. For buoyancy assisted flow the increase in the value of *N*_*t*_ decreases the local skin friction in both directions and for buoyancy opposing flow the increase in the value of *N*_*t*_ increases the local skin friction. It is evident from Figures 3 and 4 the effects of buoyancy parameter *N*_*r*_ are more prominent than thermophoresis parameter.  Figure 5 demonstrates that for both buoyancy assisted and buoyancy opposing flows the increase in the values of *N*_*b*_ increases the skin friction in both directions.

**Figure 6:**
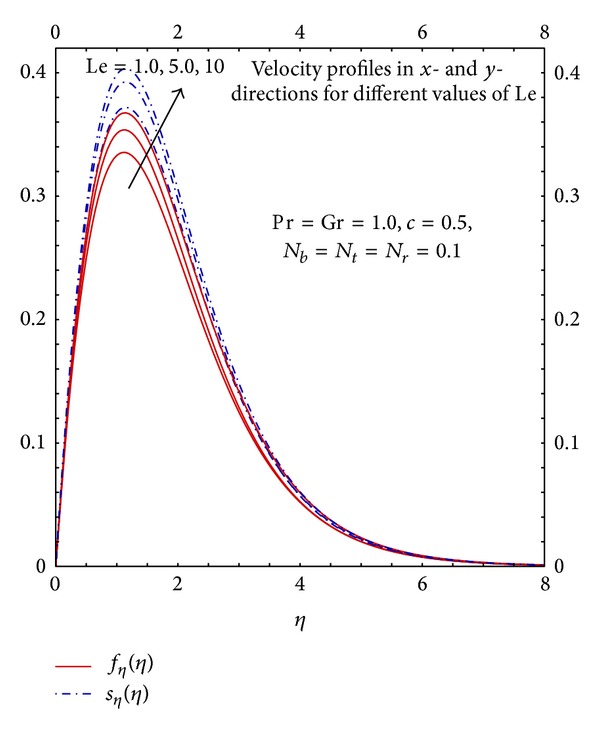
Graphs of *f*_*η*_(*η*) and *s*_*η*_(*η*) for different Le.

Figure 6 depicts the general trend of velocity profiles when *T*_*w*_ > *T*_*∞*_ and *ρ*_*p*_ > *ρ*_*f*_*∞*__. It can be seen that velocity is zero at the stagnation point. Since the temperature is higher slightly away from the wall in the ambient fluid region therefore the particles moves from cold to hot region hence the velocity tends to increase. As it goes further away the temperature difference reduces which results in the decay of the buoyancy force and flow starts decreasing. Eventually away from the wall when temperature differences are negligible the flow asymptotically achieves the free stream velocity. The behavior of increase in the values of Le on the velocity field *f*′(*η*) can be seen from Figure 6. The increase in the values of Le increases the velocity and momentum boundary layer thickness. Physically it validates the fact that increase in the values of Le increases thermal diffusivity in free convection which is due to a higher temperature therefore higher Lewis number results in higher flow field.

**Figure 7:**
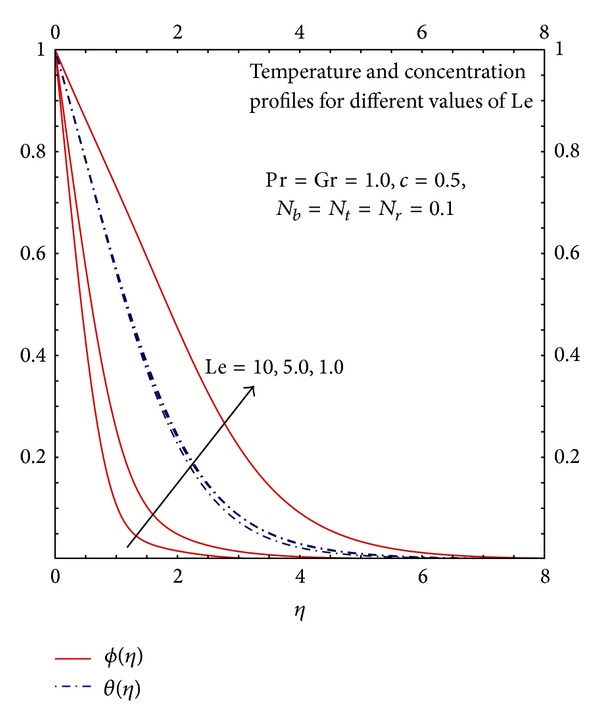
Graphs of *θ*_*η*_(*η*) and *ϕ*_*η*_(*η*) for different Le.

Figure 7 depicts the effects of dimensionless temperature and concentration profiles for buoyancy assisted flow *T*_*w*_ > *T*_*∞*_ for various values of Le. At the wall the temperature is maximum; as it moves away it is noticed the temperature reduces and asymptotically it approaches to the free stream temperature. It is observed with the increase in the values of Le the temperature and concentration profiles decreases throughout in the boundary layer. This implies that thermal boundary layer thickness decreases with the increase in the values of Le but the effect of Le is more prominent for concentration profiles. It is due to the fact the increase in the Lewis number reduces the mass diffusivity therefore heat transfer and mass transfer slow down through the fluid because the fluid conducts heat slowly relative to its volumetric heat capacity.

**Figure 8:**
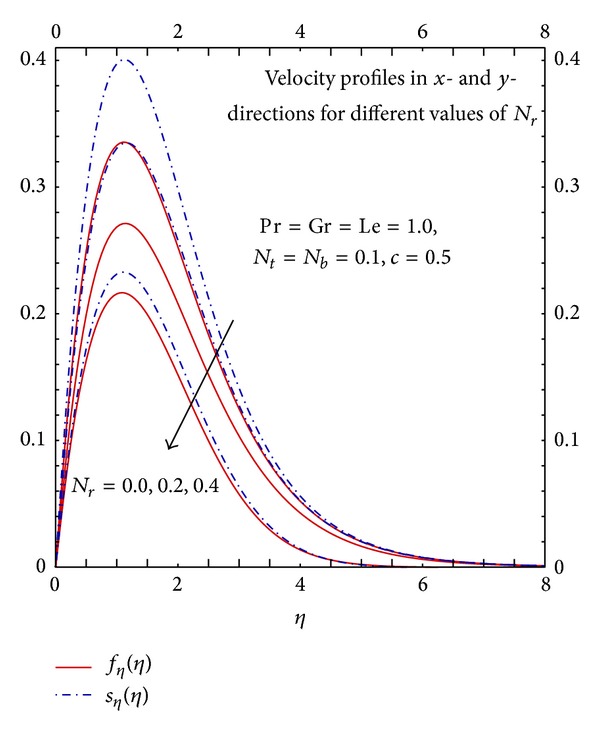
Graphs of *f*_*η*_(*η*) and *s*_*η*_(*η*) for different *N*_*r*_.

**Figure 9:**
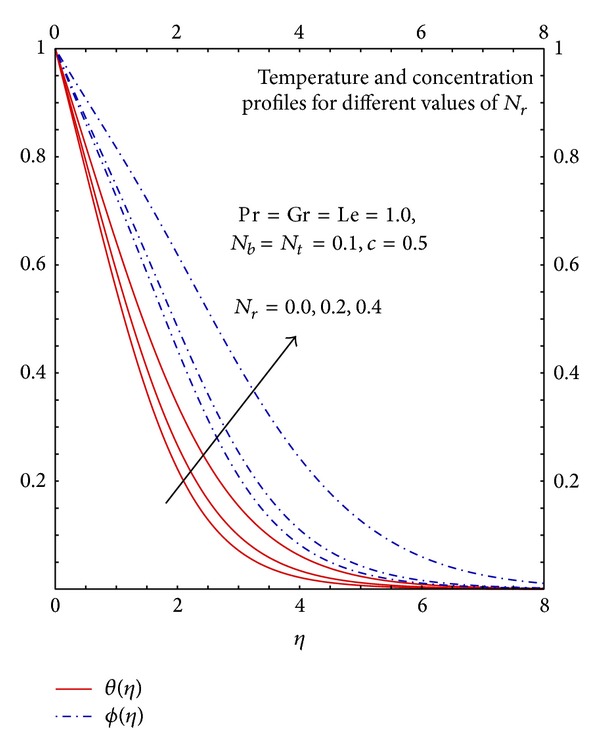
Graphs of *θ*(*η*) and *ϕ*(*η*) for different *N*_*r*_.

Analysis of Figures 8 and 9 reveals the strong effects of buoyancy coefficient *N*_*r*_ on velocity, temperature, and concentration profiles. In the absence of buoyancy parameter the density difference is zero therefore the flow is maximum; as we increase the buoyancy parameter the density difference increases which results in the decrease in the fluid flow. The increase in the buoyancy parameter increases the density difference and mass concentration difference hence it enhances the temperature and concentration profiles which shows that the effects of natural convection are significant. In free convection along with other parameters, thermophoresis parameter plays a significant role in the flow, heat transfer, and mass transfer properties.

**Figure 10:**
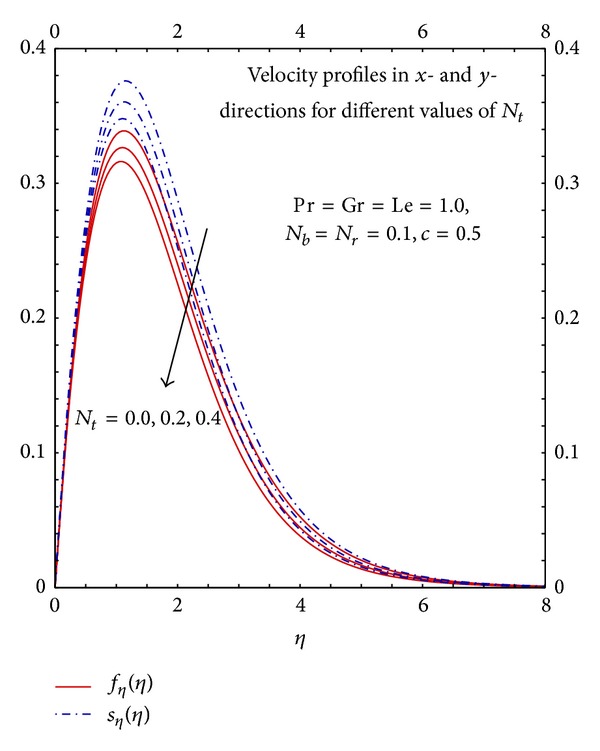
Graphs of *f*_*η*_(*η*) and *s*_*η*_(*η*) for different *N*_*t*_.

Figure 10 illustrates the fluid flow is reduced with the increase in the values of *N*_*t*_. It is clear the increase in the values of *N*_*t*_ increases the temperature difference which results in the decrease in the fluid flow hence nanoparticles in the fluid opposes the transport phenomena through the parameter *N*_*t*_. Further, it can be seen the highest value of the velocity occur in the ambient fluid close to the surface but not at the surface.

**Figure 11:**
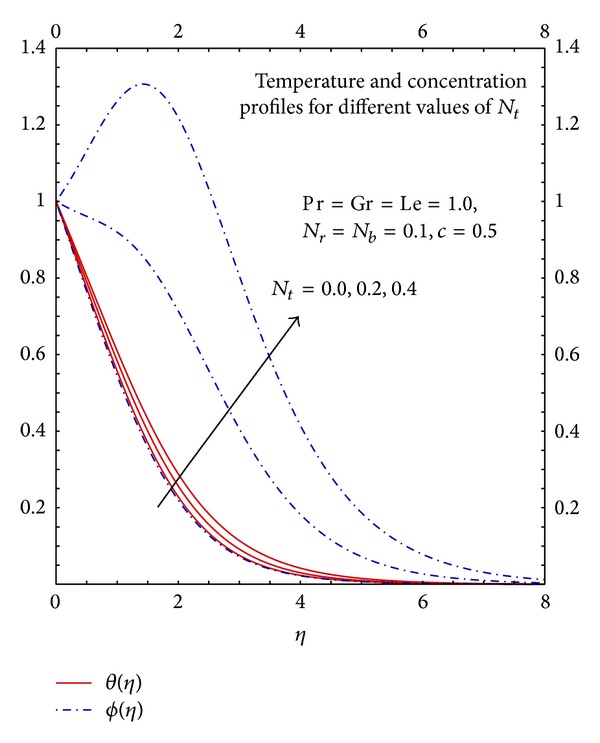
Graphs of *θ*(*η*) and *ϕ*_*η*_ for different *N*_*t*_.

Figure 11 illustrates the effects of *N*_*t*_ on the fluid temperature and concentration profiles. The increase in the values of *N*_*t*_ increases the temperature and concentration profiles hence the nanoparticles enhance heat and mass transfer. It is observed that the effects of *N*_*t*_ are more pronounced for concentration profiles.

**Figure 12:**
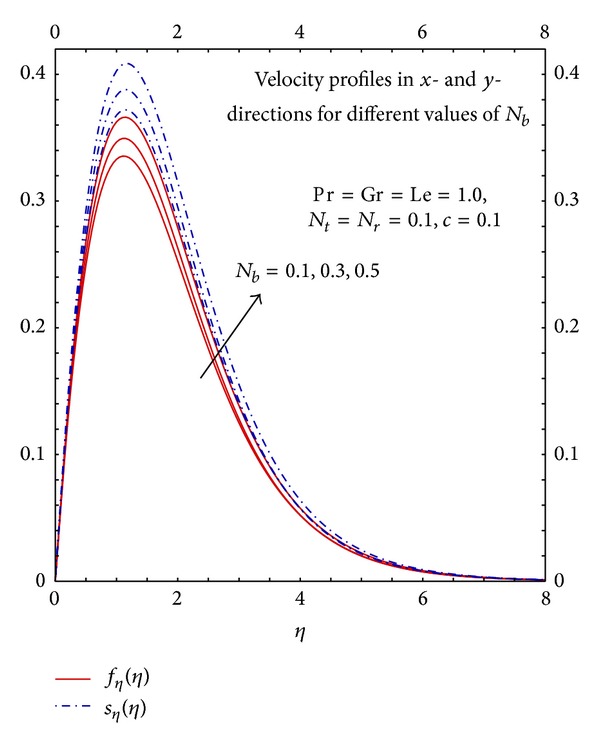
Graphs of *f*_*η*_(*η*) and *S*_*η*_(*η*) for different *N*_*b*_.

**Figure 13:**
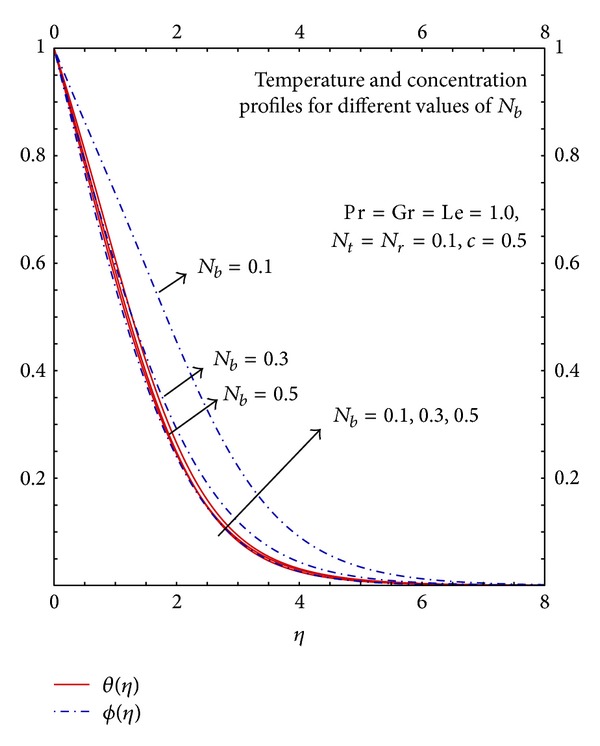
Graphs of *θ*(*η*) and *ϕ*(*η*) for different *N*_*b*_.

Figure 12 depicts that the increase in the values of *N*_*b*_ increases the flow because the increase in Brownian motion parameter will increase the mass diffusivity which enhances the momentum transfer.  Figure 13 depicts that the increase in Brownian motion parameter increases the mass diffusivity which enhances the heat transfer process therefore temperature profiles increases with the increase in the values of *N*_*b*_. The increase in the values of *N*_*b*_ reduces thermal diffusivity therefore the mass transfer will decrease hence the increase in the values of *N*_*b*_ decreases the concentration profiles.

## 5. Conclusions

The problem of steady flow and heat transfer of free convection boundary layer flow in the stagnation-point region in the presence of nanoparticles is studied analytically. The governing equations are nondimensionalized using proper nondimensional quantities. The resulting boundary value problem is solved by optimal homotopy analysis method. A new kind of total averaged squared residual error is used to obtain the optimal values of the convergence-control parameters. The procedure to obtain global convergence control parameters is defined in detail. The effects of the various values of the pertinent parameters *c*, *N*_*b*_, *N*_*t*_, *N*_*r*_, and Le on the fluid velocity, temperature profile, and concentration profile are illustrated through graphs. We may extract some important findings from our results.

- The increase in the values of *c* reduces the skin friction.
- The increase in the values of *c* increases the Local Nusselt number and local Sherwood number therefore it enhances heat and mass transfer.
- The increase in the values of Le and *N*_*b*_ will enhance flow in both *x*- and *y*-directions.
- The effect of the increase in the values of *N*_*r*_ and *N*_*t*_ is to decelerate the flow.
- The increase in the values of *N*_*r*_, *N*_*b*_, and *N*_*t*_ increases the temperature while quite the opposite is true for Le.
- The effect of the increase in the values of Le and *N*_*b*_ is to decrease the concentration profiles.
- The effect of the increase in the values of *N*_*r*_ and *N*_*t*_ is to increase the concentration profiles.

We may conclude the flow and heat transfer properties of free convection boundary layer flow in the stagnation-point region in the presence of nanoparticles can be controlled by changing the quantity of the physical parameters. Hence OHAM is very effective method to solve strongly nonlinear problems.

## Figures and Tables

**Table 1:** Global optimal convergence control parameters at different orders of approximations for Pr = Gr = Le = 1.0, *N*_*b*_ = *N*_*t*_ = *N*_*r*_ = 0.1.

*k*	*c*_0_^*f*^	*c*_0_^*s*^	*c*_0_^*θ*^	*c*_0_^*ϕ*^	*E*_*k*_^*t*^
1	−1.05	−2.14	−1.02	−0.69	0.59
3	−1.14	−1.89	−1.02	−1.25	0.15
5	−1.56	−1.86	−1.17	−1.36	0.05

**Table 2:** Averaged squared residual errors using *c*_0_^*f*^ = −1.56, *c*_0_^*s*^ = −1.86, *c*_0_^*θ*^ = −1.17, and *c*_0_^*ϕ*^ = −1.36 where Pr = Gr = Le = 1.0, *N*_*b*_ = *N*_*t*_ = *N*_*r*_ = 0.1, and *c* = 0.5.

*k*	60	80	100
*E*_*k*_^*f*^	0.000014	6.98 × 10^−6^	3.96 × 10^−6^
*E*_*k*_^*s*^	0.000010	5.14 × 10^−6^	2.91 × 10^−6^
*E*_*k*_^*θ*^	0.000015	5.01 × 10^−6^	2.23 × 10^−6^
*E*_*k*_^*ϕ*^	0.000013	6.45 × 10^−6^	3.70 × 10^−6^
*E*_*k*_^*t*^	0.000054	0.000023	0.000012

## Appendices

### A. Homotopy-Based Approach

We apply the optimal homotopy analysis method to solve the system of nonlinear ordinary differential equations (7)–(10) with boundary conditions (13). In the framework of the HAM the solutions for *f*(*η*), *s*(*η*), *θ*(*η*), and *ϕ*(*η*) can be expressed explicitly by an infinite number of subfunctions in the following form:

(A.1) $$\begin{array}{c} f\left(\eta \right)=\overset{+\infty }{\underset{k=0}{\sum }}f_{k}\left(\eta \right),\;\;s\left(\eta \right)=\overset{+\infty }{\underset{k=0}{\sum }}s_{k}\left(\eta \right),\\ \theta \left(\eta \right)=\overset{+\infty }{\underset{k=0}{\sum }}\theta_{k}\left(\eta \right),\;\;\phi \left(\eta \right)=\overset{+\infty }{\underset{k=0}{\sum }}\phi_{k}\left(\eta \right). \end{array}$$

From physical points of view, it is clear that all the solutions should be in the form as given below; therefore

(A.2) $$\begin{array}{c} f\left(\eta \right)=A_{0,0}^{k}+\overset{+\infty }{\underset{k=0}{\sum }}\;\overset{+\infty }{\underset{j=1}{\sum }}\;\overset{+\infty }{\underset{i=0}{\sum }}A_{i,j}^{k}\eta^{i}\exp\left(-j\eta \right),\\ \theta \left(\eta \right)=\overset{+\infty }{\underset{k=0}{\sum }}\;\overset{+\infty }{\underset{j=1}{\sum }}\;\overset{+\infty }{\underset{i=0}{\sum }}C_{i,j}^{k}\eta^{i}\exp\left(-j\eta \right),\\ s\left(\eta \right)=B_{0,0}^{k}+\overset{+\infty }{\underset{k=0}{\sum }}\;\overset{+\infty }{\underset{j=1}{\sum }}\;\overset{+\infty }{\underset{i=0}{\sum }}B_{i,j}^{k}\eta^{i}\exp\left(-j\eta \right),\\ \phi \left(\eta \right)=\overset{+\infty }{\underset{k=0}{\sum }}\;\overset{+\infty }{\underset{j=1}{\sum }}\;\overset{+\infty }{\underset{i=0}{\sum }}D_{i,j}^{k}\eta^{i}\exp\left(-j\eta \right), \end{array}$$

where *A*_*i*,*j*_^*k*^, *B*_*i*,*j*_^*k*^, *C*_*i*,*j*_^*k*^, and *D*_*i*,*j*_^*k*^ are constant coefficients to be determined. In the HAM, one has great freedom on the choice of the auxiliary linear operator. Thus, we can choose auxiliary linear operators as

(A.3) $$\begin{array}{c} L_{1}\left[u\left(\eta :q\right)\right]=\frac{{\text{d}}^{3}u}{\text{d}\eta^{3}}-\frac{\text{d}u}{\text{d}\eta },\\ L_{2}\left[v\left(\eta :q\right)\right]=\frac{{\text{d}}^{2}v}{\text{d}\eta^{2}}-v, \end{array}$$

which satisfy the following properties:

(A.4) $$\begin{array}{c} L_{1}\left[C_{1}\exp\left(-\eta \right)+C_{2}\exp\left(\eta \right)+C_{3}\right]=0,\\ L_{2}\left[C_{9}\exp\left(-\eta \right)+C_{10}\exp\left(\eta \right)\right]=0. \end{array}$$

The corresponding auxiliary linear operator for *f*(*η*) and *s*(*η*) is *L*_1_ and *L*_2_ corresponds to *θ*(*η*) and *ϕ*(*η*). The auxiliary function *H*_*i*_ (*i* = 1–3) is chosen as

(A.5) $$\begin{array}{c} H_{i}=\exp\left(-\eta \right). \end{array}$$

In HAM, we also have freedom to choose the initial approximations. Note that, all the initial approximations need to satisfy the boundary conditions (14). Without loss of generality, we set

(A.6) $$\begin{array}{c} f_{0}\left(\eta \right)=\frac{1}{2}-e^{-\eta }+\frac{1}{2}e^{-2\eta },\\ s_{0}\left(\eta \right)=\frac{1}{5}\left(\frac{1}{2}-e^{-\eta }+\frac{1}{2}e^{-2\eta }\right),\\ \theta_{0}\left(\eta \right)=\exp\left(-\eta \right),\\ \phi_{0}\left(\eta \right)=\exp\left(-\eta \right). \end{array}$$

Thus, in the frame of HAM the *k*th order approximation *f*_*k*_(*η*), *s*_*k*_(*η*), *θ*_*k*_(*η*), and *ϕ*_*k*_(*η*) can be obtained using the following recursive formulae:

(A.7) fk(η)=χkfk−1(η)+fk∗(η)+C1k+C2keη+C3ke−η,sk(η)=χksk−1(η)+sk∗(η)+C4k+C5keη+C6ke−η,θk(η)=χkθk−1(η)+θk∗(η)+C7k+C8ke−η,ϕk(η)=χkϕk−1(η)+ϕk∗(η)+C9k+C10ke−η,

where

(A.8) $$\begin{array}{c} \chi_{m}=\{\begin{array}{cc} 0, & m\leq 1\\ 1, & m>1. \end{array} \end{array}$$

*C*_1_^*k*^, *C*_2_^*k*^, *C*_3_^*k*^, *C*_4_^*k*^, *C*_5_^*k*^, *C*_6_^*k*^, *C*_7_^*k*^, *C*_8_^*k*^, *C*_9_^*k*^, and *C*_10_^*k*^ are integral constants determined as

(A.9) $$\begin{array}{c} C_{1}^{k}=-f_{k}^{*\prime }\left(0\right)-f_{k}^{*}\left(0\right),\;\;C_{2}^{k}=0,\;\;C_{3}^{k}=f_{k}^{*\prime }\left(0\right),\\ C_{4}^{k}=-s_{k}^{*\prime }\left(0\right)-s_{k}^{*}\left(0\right),\;\;C_{5}^{k}=0,\;\;C_{6}^{k}=s_{k}^{*\prime },\\ C_{7}^{k}=0,\;\;C_{8}^{k}=1,\;\;C_{9}^{k}=0,\;\;C_{10}^{k}=1. \end{array}$$

and *f*_*k*_*(*η*), *s*_*k*_*(*η*), *θ*_*k*_*(*η*), and *ϕ*_*k*_*(*η*) are particular solutions defined by

(A.10) $$\begin{array}{c} f_{k}^{*}\left(\eta \right)=L_{1}^{-1}\left[c_{0}^{f}R_{k}^{f}\right],\;\;s_{k}^{*}\left(\eta \right)=L_{1}^{-1}\left[c_{0}^{f}R_{k}^{s}\right],\\ \theta_{k}^{*}\left(\eta \right)=L_{2}^{-1}\left[c_{0}^{\theta }R_{k}^{\theta }\right],\;\;\phi_{k}^{*}\left(\eta \right)=L_{2}^{-1}\left[c_{0}^{\phi }R_{k}^{\phi }\right]. \end{array}$$

*c*_0_^*f*^, *c*_0_^*s*^, *c*_0_^*θ*^, and *c*_0_^*ϕ*^ are convergence-control parameters, *R*_*k*_^*f*^, *R*_*k*_^*s*^, *R*_*k*_^*θ*^, and *R*_*k*_^*ϕ*^ are defined based on (8)–(13) for details see Liao [30]. Consider

(A.11) Rkf=fk−1′′′+∑i=0k−1fi′′fk−1−i+c∑i=0k−1fi′′sk−1−i−∑i=0k−1fi′fk−1−i′+θk−1−NrPr Grϕk−1,Rks=sk−1′′′+∑i=0k−1si′′fk−1−i+c∑i=0k−1si′′sk−1−i−c∑i=0k−1fi′fk−1−i′+θk−1−NrPr Grϕk−1,Rkθ=1Prθk−1′′+∑i=0k−1θi′fk−1−i+c∑i=0k−1θi′sk−1−i+NbPr∑i=0k−1θi′ϕk−1−i′+NtPr∑i=0k−1θi′θk−1−i′,Rkϕ=ϕk−1′′+NtNb+Le Pr(∑i=0k−1ϕi′fm−1−i+c∑i=0k−1ϕi′sk−1−i).

Using the above recursive formulae, we can get the explicit analytical approximations of *f*_*k*_(*η*), *s*_*k*_(*η*), *θ*_*k*_(*η*), and *ϕ*_*k*_(*η*) for *k* = 1,2, 3,….

### B. Formulation of Explicit Solutions

The corresponding coefficients in (A.2) are obtained by means of HAM as

(B.1) f(η)=A0,0k+∑j=1+∞ ∑i=0+∞Ai,jkηiexp⁡(−jη),θ(η)=∑j=1+∞ ∑i=0+∞Ci,jkηiexp⁡(−jη),s(η)=B0,0k+∑j=1+∞ ∑i=0+∞Bi,jkηiexp⁡(−jη),ϕ(η)=∑j=1+∞ ∑i=0+∞Di,jkηiexp⁡(−jη),A0,0k=χkA0,0k−1+∑j=2k+1 ∑q=1k+1Γq,jk[(j−1)ωj,0q−1λωj,1q]−∑q=0k1λΔq,1kω1,1q+∑j=2k+1(j−1)Γ0,jkωj,00,A0,1k=χkσ0,1k−1A0,1k−1+∑j=2k+1 ∑q=1k+11λΓq,jkωj,1q+∑q=0k1λΔq,1kω1,1q−∑j=2k+1 ∑q=0k+1jΓq,jkωj,0q,A0,jk=0, (2≤j≤k+1),Ai,1k=χkσi,1k−1A0,1k−1+∑q=ik+1Δq,1kω1,iq, (1≤i≤k+1),Ai,jk=χkσi,jk−1A0,1k−1+∑q=ik+1Γq,jkωj,1q, (1≤i≤k+1,(2≤j≤k+1)),B0,0k=χkB0,0k−1+∑j=2k+1 ∑q=1k+1Θq,jk[(j−1)ωj,0q−1λωj,1q]−∑q=0k1λΛq,1kω1,1q+∑j=2k+1(j−1)Θ0,jkωj,00,B0,1k=χkσ0,1k−1B0,1k−1+∑j=2k+1 ∑q=1k+11λΘq,jkωj,1q+∑q=0k1λΛq,1kω1,1q−∑j=2k+1 ∑q=0k+1jΘq,jkωj,0q,B0,jk=0, (2≤j≤k+1),Bi,1k=χkσi,1k−1B0,1k−1+∑q=ik+1Θq,1kω1,iq, (1≤i≤k+1),C0,1k=χkσ0,1k−1C0,0k−1−∑j=2k+1 ∑q=0k+1Ψq,jkδj,0q,Ci,1k=χkδi,1k−1Ci,1k−1−∑q=max⁡⁡[0,i−1]k+1Ωq,1kδ1,iq (1≤i≤k+1),C0,jk=0, (2≤j≤k+1),Ci,jk=χkδi,jk−1Ci,jk−1+∑q=ik+1Ωq,jkδj,iq (1≤i≤k+1,2≤j≤k+1),D0,1k=χkσ0,1k−1D0,0k−1−∑j=2k+1 ∑q=0k+1Φq,jkδj,0q,Di,1k=χkδi,1k−1Di,1k−1−∑q=max⁡⁡[0,i−1]k+1Υq,1kδ1,iq (1≤i≤k+1),D0,jk=0, (2≤j≤k+1),Di,jk=χkδi,jk−1Di,jk−1+∑q=ik+1Φq,jkδj,iq (1≤i≤k+1,2≤j≤k+1),

with

(B.2) $$\begin{array}{c} \sigma_{i,j}^{k}=\{\begin{array}{cc} 1, & 0\leq i\leq k+1,\;\;1\leq j\leq k+1\\ 0, & \text{otherwise}, \end{array}\\ \omega_{n,j}^{q}=\{\begin{array}{cc} \overset{q+1}{\underset{i=j}{\sum }}\frac{\delta_{n,j}^{q}}{\lambda^{i-j+1}}, & 0\leq j\leq q+1,\;\;n=1,\\ \overset{q}{\underset{i=j}{\sum }}\frac{\delta_{n,j}^{q}}{{\left(n\lambda \right)}^{i-j+1}}, & 0\leq j\leq q+1,\;\;n>1,\\ 0, & \text{otherwise}, \end{array}\\ \delta_{n,j}^{q}=\{\begin{array}{cc} \frac{q!}{j!}\frac{1}{{\left(2\lambda \right)}^{q}-j+2},\;\;0\leq j\leq q,\;\;n=1, & \\ \frac{1}{2\lambda \left(q+1\right)},\;\;\;\;\;\;0\leq j\leq q+1,\;\;n>1, & \\ \frac{1}{2\lambda }\frac{q!}{j!}\left\{\frac{1}{{\left[\left(n+1\right)\lambda \right]}^{q}-j+1}\right\} & \\ \;-\frac{q!}{j!}\left\{\frac{1}{{\left[\left(n-1\right)\lambda \right]}^{q}-j+1}\right\}, & \\ \;\;\;\;\;\;\;\;\;0\leq j\leq q,\;\;n>1 & \\ 0,\;\;\;\;\;\;\;\;\;j=q+1,\;\;n>1. & \end{array} \end{array}$$

The functions in the above recurrence formulae are given by

(B.3) Γi,jk=c0f(A3i,jk−1+Ci,jk−1−NrPr GrDi,jk−1+Δ1i,jk+Δ2i,jk  +Δ3i,jk+Γ1i,jk+Γ2i,jk),Θi,jk=c0s(B3i,jk−1+Ci,jk−1−NrPr GrDi,jk−1+Δ4i,jk+Δ5i,jk  +Δ6i,jk+Γ3i,jk+Γ4i,jk),Ψi,jk=c0θ(1PrC3i,jk−1+Δ7i,jk+Δ8i,jk+Δ9i,jk  +Δ10i,jk+Γ5i,jk+Γ6i,jk),Φi,jk=c0ϕ(Di,jk−1+NtNbCi,jk−1+Δ11i,jk+Δ12i,jk  +Γ7i,jk+Γ8i,jk),Δi,1k=c0f(A3i,1k−1+Ci,1k−1−NrPrGrDi,1k−1+Γ1i,1k+Γ2i,1k),Λi,1k=c0s(B3i,1k−1+Ci,1k−1−NrPr GrDi,1k−1+Γ3i,1k+Γ4i,1k),Ωi,1k=c0θ(C3i,1k−1+Γ5i,1k+Γ6i,1k),Υi,1k=c0ϕ(D3i,1k−1+Γ7i,1k+Γ8i,1k),

where *c*_*m*_^*f*^, *c*_*m*_^*s*^, *c*_*m*_^*θ*^, and *c*_*m*_^*ϕ*^ are the HAM auxiliary parameters, and

(B.4) A1i,jk=(i+1)σi+1,jkAi+1,jk−jλσi+1,jkAi,jk,B1i,jk=(i+1)σi+1,jkBi+1,jk−jλσi+1,jkBi,jk,C1i,jk=(i+1)σi+1,jkCi+1,jk−jλσi+1,jkCi,jk,D1i,jk=(i+1)σi+1,jkDi+1,jk−jλσi+1,jkDi,jk,A2i,jk=(i+2)(i+1)σi+2,jkAi+2,jk−2jλ(i+1)σi+1,jkAi+1,jk+(jλ)2(i+1)σi,jkAi,jk,B2i,jk=(i+2)(i+1)σi+2,jkBi+2,jk−2jλ(i+1)σi+1,jkBi+1,jk+(jλ)2(i+1)σi,jkBi,jk,C2i,jk=(i+2)(i+1)σi+2,jkCi+2,jk−2jλ(i+1)σi+1,jkCi+1,jk+(jλ)2(i+1)σi,jkCi,jk,D2i,jk=(i+2)(i+1)σi+2,jkDi+2,jk−2jλ(i+1)σi+1,jkDi+1,jk+(jλ)2(i+1)σi,jkDi,jk,A3i,jk=(i+3)(i+2)(i+1)σi+3,jkAi+3,jk−3jλ(i+2)(i+1)σi+2,jkAi+2,jk+ ß(i+1)(jλ)2σi+1,jkAi+1,jk−(jλ)3σi,jkAi,jk,B3i,jk=(i+3)(i+2)(i+1)σi+3,jkBi+3,jk−3jλ(i+2)(i+1)σi+2,jkBi+2,jk+ ß(i+1)(jλ)2σi+1,jkBi+1,jk−(jλ)3σi,jkBi,jk,Δ1i,jk=∑n=0k−1 ∑s=max⁡{1,j−n−1}min⁡{k−n,j−1} ∑r=max⁡{0,i−n−1}min⁡{k−n,i}σr,sk−1−nAr,sk−1−nA2i−r,j−sn,Δ2i,jk=c∑n=0k−1 ∑s=max⁡{1,j−n−1}min⁡{k−n,j−1} ∑r=max⁡{0,i−n−1}min⁡{k−n,i}σr,sk−1−nBr,sk−1−nA2i−r,j−sn,Δ3i,jk=−1∑n=0k−1 ∑s=max⁡{1,j−n−1}min⁡{k−n,j−1} ∑r=max⁡{0,i−n−1}min⁡{k−n,i}σr,sk−1−nA1r,sk−1−nA1i−r,j−sn,Δ4i,jk=∑n=0k−1 ∑s=max⁡{1,j−n−1}min⁡{k−n,j−1} ∑r=max⁡{0,i−n−1}min⁡{k−n,i}σr,sk−1−nAr,sk−1−nB2i−r,j−sn,Δ5i,jk=c∑n=0k−1 ∑s=max⁡{1,j−n−1}min⁡{k−n,j−1} ∑r=max⁡{0,i−n−1}min⁡{k−n,i}σr,sk−1−nBr,sk−1−nB2i−r,j−sn,Δ6i,jk=−c∑n=0k−1 ∑s=max⁡⁡{1,j−n−1}min⁡⁡{k−n,j−1} ∑r=max⁡⁡{0,i−n−1}min⁡⁡{k−n,i}σr,sk−1−nB1r,sk−1−nB1i−r,j−sn,Δ7i,jk=∑n=0k−1 ∑s=max⁡{1,j−n−1}min⁡{k−n,j−1} ∑r=max⁡{0,i−n−1}min⁡{k−n,i}σr,sk−1−nAr,sk−1−nC1i−r,j−sn,Δ8i,jk=c∑n=0k−1 ∑s=max⁡{1,j−n−1}min⁡{k−n,j−1} ∑r=max⁡{0,i−n−1}min⁡{k−n,i}σr,sk−1−nBr,sk−1−nC1i−r,j−sn,Δ9i,jk=NbPr∑n=0k−1 ∑s=max⁡⁡{1,j−n−1}min⁡⁡{k−n,j−1} ∑r=max⁡⁡{0,i−n−1}min⁡⁡{k−n,i}σr,sk−1−nC1r,sk−1−nD1i−r,j−sn,Δ10i,jk=NtPr∑n=0k−1 ∑s=max⁡⁡{1,j−n−1}min⁡⁡{k−n,j−1} ∑r=max⁡⁡{0,i−n−1}min⁡⁡{k−n,i}σr,sk−1−nC1r,sk−1−nC1i−r,j−sn,Δ11i,jk=Le·Pr∑n=0k−1∑s=max⁡{1,j−n−1}min⁡{k−n,j−1}∑r=max⁡{0,i−n−1}min⁡{k−n,i}σr,sk−1−nAr,sk−1−nD1i−r,j−sn,Δ12i,jk   =Le·Pr·c      ×∑n=0k−1∑s=max⁡{1,j−n−1}min⁡{k−n,j−1}∑r=max⁡{0,i−n−1}min⁡{k−n,i}σr,sk−1−nBr,sk−1−nD1i−r,j−sn,Γ1i,jk=∑n=max⁡{j−1,i−1}k−1A0,0k−1−nA2i,jn,Γ2i,jk=∑n=max⁡{j−1,i−1}k−1B0,0k−1−nA2i,jn,Γ3i,jk=∑n=max⁡{j−1,i−1}k−1A0,0k−1−nB2i,jn,Γ4i,jk=∑n=max⁡{j−1,i−1}k−1B0,0k−1−nB2i,jn,Γ5i,jk=∑n=max⁡{j−1,i−1}k−1A0,0k−1−nC1i,jn,Γ6i,jk=∑n=max⁡{j−1,i−1}k−1B0,0k−1−nC1i,jn,Γ7i,jk=∑n=max⁡{j−1,i−1}k−1A0,0k−1−nD1i,jn,Γ8i,jk=∑n=max⁡{j−1,i−1}k−1B0,0k−1−nD2i,jn.

Therefore by setting *A*_0,0_^0^, *A*_0,1_^0^, *B*_0,0_^0^, *B*_0,1_^0^, and *C*_0,1_^0^, all coefficients can be determined successively in the order *k* = 1,2, 3,….
